# The profile of long-stay patients in a psychiatric hospital in KwaZulu-Natal, South Africa

**DOI:** 10.4102/sajpsychiatry.v31i0.2358

**Published:** 2025-01-24

**Authors:** Siziphiwe Y. Myeni, Vuyokazi Ntlantsana, Andrew Tomita, Ugochukwu S. Aguwa, Reyanta Bridgmohun, Sinethemba Shabalala

**Affiliations:** 1 Discipline of Psychiatry, College of Health Sciences, University of KwaZulu-Natal, Durban, South Africa; 2 Centre for Rural Health, School of Nursing and Public Health, University of KwaZulu-Natal, Durban, South Africa; 3 Department of Anatomy, Faculty of Basic Medical Sciences, Nnamdi Azikiwe University, Awka, Nigeria; 4 Department of Psychiatry, Faculty of Basic Medical Sciences, Nnamdi Azikiwe University, Awka, Nigeria

**Keywords:** profile, psychiatry, long-stay, inpatient, multiple admissions, involuntary admissions, psychotic disorders, length of stay

## Abstract

**Background:**

South African psychiatric hospitals’ inpatient average length of stay is approximately 220 days. Inpatient care accounts for over 80% of the mental national healthcare budget. However, there is limited research on factors associated with length of stay (LOS) in tertiary psychiatric hospitals.

**Aim:**

To determine the threshold for long-stay and describe the socio-demographic and clinical profile of long-stay patients admitted to a tertiary psychiatric hospital.

**Setting:**

Townhill Hospital: a tertiary psychiatric hospital in Pietermaritzburg, KwaZulu-Natal, South Africa.

**Methods:**

A retrospective review of clinical records of admissions between January 2019 and January 2020 was conducted. Information on LOS, sociodemographic and clinical factors was collected. The interquartile range (IQR) of LOS in days was calculated, with patients above 75th percentile classified as long-stay patients.

**Results:**

In total, 326 records were included. The 75th percentile LOS was 120 days. Eighty-three patients (25.46%) had a stay of 120 days or longer. The median LOS was 73 (IQR 49–120) days. Factors associated with long-stay included being male (*p* < 0.001), a psychotic disorder diagnosis (*p* = 0.019), receiving a disability grant (*p* = 0.050), involuntary admission (*p* = 0.010) and multiple readmissions (*p* = 0.010).

**Conclusion:**

Psychotic disorders and associated factors are key contributors to long-stay hospitalisations.

**Contribution:**

This study highlights the burden of inpatient care for psychotic disorders and the need for interventions that optimise care and promote remission. To reduce the economic impact of prolonged hospitalisations, early intervention and enhanced community-based mental healthcare services focused on psychotic disorders are recommended.

## Introduction

By 2019, one in eight people around the world was living with a mental disorder and the prevalence of mental disorders continues to rise.^1,2^ With a mental healthcare gap (the percentage of people requiring treatment but, for various reasons, not receiving it),^3^ of up to 85%, and a third of financial expenditure on standalone mental hospitals, health systems are not responding adequately to the burden of mental health disorders.^4^ To close this significant gap and improve access to care, the World Health Organization (WHO) recommends redirecting funding towards community-based services as a means to foster better and more cost-effective interventions.^4^ According to Chisholm, Saxena and Van Ommeren,^5^ in order for a country to have a comprehensive mental health system, it needs to allocate between 5% and 10% of the total health budget to mental health. South African’s expenditure on mental healthcare is estimated at 5% of the total public healthcare budget, but six out of nine provinces spend less than this amount.^1^ Furthermore, 86% of this budget has been reported to have been spent on inpatient care and almost half of that at tertiary hospitals.^1^

By 2019, the average length of stay (ALOS) for inpatient care for mental health conditions in South African hospitals was 157 days, up to eight times longer than inpatient admissions for all other health conditions, and 93% of these admissions were adults aged 18 years and older.^1^ The National Mental Health Policy Framework and Strategic Plan 2023–2030 outlines plans to downscale specialist mental healthcare facilities and upscale community-based care facilities, in line with WHO recommendations.^6^ The ALOS in specialised psychiatric hospitals in KwaZulu-Natal (KZN) is reported at 399 days.^7^ This figure includes patients requiring long-term chronic care at specialised institutions. Fisher, Barreira^8^ in their study of patients who had exceeded 3 years of stay in a contemporary psychiatric hospital – a hospital focused on improving quality of life, functioning and skill development rather than just addressing clinical symptoms^9^ – found that although some patients were fit for discharge into long-term care, these facilities were not always available and patients therefore spent time in resource intense psychiatric hospitals.

The ALOS serves as an indicator of efficient healthcare delivery, with shorter hospital stays associated with reduction in cost allowing for a shift in care to less expensive community-based services.^10,11^ In psychiatric care, long hospital stay has been associated with higher cost, negative outcomes including stigma, deterioration in social functioning and low satisfaction, which affects attitudes towards future health-seeking behaviour.^2,12,13,14,15,16^ Furthermore, long-stay at specialist psychiatric hospitals limit bed availability, causing delays in transferring other patients from non-specialised units who require specialised care.^17^ In the United Kingdom (UK), mental health policy recommends short stays of less than 28 days for individuals with mental illness, as this may increase the likelihood of employment and reduce a revolving door pattern of admissions.^18^

Although there is a shared consensus that there has been a decline in the length of stay (LOS) in psychiatric inpatient units, there are still instances where patients endure lengthy hospital stays.^2,19^ Additionally, there is currently no clear consensus on the definition of long-stay in literature.^2,12,19,20,21,22,23,24^ Long-stay hospitalisation has been described as longer than 2 months; others have described it as a stay of longer than 6 months.^23,25^ A better understanding of the problem of long-stay and delineating the factors associated with it is an important aspect for comprehensive healthcare planning and in identifying areas that need to be the target of interventions in mental healthcare.

A number of socio-demographic and clinical factors have been identified as being associated with LOS such as gender,^2,19,20,22,23^ age,^12,22^ marital status^2^ and employment.^19,22^ However, even among these studies, the evidence has been inconsistent. A number of studies found a diagnosis of psychosis and an involuntary admission status to be associated with longer LOS.^2,12,19,22,24^ Others have reported the presence of a comorbid medical condition to be associated with longer LOS.^20,21,22,23^ The conflicting data regarding factors associated with long-stay are linked to the lack of consensus about the duration of long-stay and that LOS may be determined by contextual factors such as resources and the differences in how mental healthcare systems are organised. The authors are not aware of any study describing long-stay and the associated factors in a tertiary or specialised psychiatric hospital in KwaZulu-Natal.

### Aim

This study aimed to determine the threshold for ‘long-stay’ and to describe the socio-demographic and clinical profile of long-stay patients admitted to Townhill Hospital, a tertiary psychiatric hospital in KwaZulu-Natal, South Africa (SA).

## Research methods and design

### Study design

This is a retrospective chart review of clinical records of patients admitted at Townhill Hospital, a 280-bed tertiary psychiatric hospital located in uMgungundlovu (Pietermaritzburg), KwaZulu-Natal, SA. The hospital provides specialist tertiary-level services for a population of approximately 1 million people and serves as a referral hospital for regional and district hospital patients that require specialised care and rehabilitation.^26^

### Study population and sampling strategy

Data from clinical records of patients admitted during the period of 01 January 2019 – 31 January 2020 who were aged 18 years and older were included. This period was chosen to avoid the influence of the coronavirus disease 2019 (COVID-19) pandemic. Data extraction was conducted by the lead author (SM) over a 2-month period, from 01 October 2022 to 30 November 2022. Patients admitted during the study period but not discharged by data collection date were excluded from the study, as their duration of stay could not be ascertained. This exclusion ensured that the study focused solely on patients who could and were discharged based on the treating clinician’s assessment that clinical management had been optimised, rather than those still hospitalised beyond the data collection date.

A sample size of at least 323 medical records was needed to provide sufficient power for the study to determine associations. This was based on a sample size calculation for infinite populations at a confidence interval of 95%, an error margin of 5% and an assumed proportion of 0.3.^27^

### Data collection

A structured data collection tool was developed by the researchers to collect information on demographic, social and clinical information from the clinical records.

The data collected from hard copy clinical records was captured onto an electronic REDCap database.^28^ This is a secure, password-protected web-based research database hosted by the University of KZN.

### Data analysis

Data analysis was conducted using the 15.1 version of Stata. Descriptive statistics are presented using frequencies and percentages to summarise results. The interquartile range (IQR) of the LOS was determined based on a method used by Krell, Girotti and Dimick,^29^ patients whose LOS was at or above the 75th percentile were classified as long-stay. In this study, LOS below the 75th percentile will be regarded as short-intermediate stay (SIS). To test for associations between categorical variables, Pearson’s Chi-squared tests was used with Fisher’s exact test used where appropriate. Wilcoxon rank-sum tests were used to test for association between asymmetrical continuous and binary data. A regression model controlling for clinical and sociodemographic variables affecting LOS was created. The level of significance was set at 0.05.

### Ethical considerations

Permission to access medical records was received from KZN Department of Health and Townhill Hospital ethics committee before commencing data collection. Ethical approval to conduct the study was obtained from the Biomedical Research Ethics Committee of the University of KZN (reference number BREC/00003852/2022).

## Results

Of the 329 clinical records of patients admitted during the study period, three were excluded because of missing data; therefore a total of 326 were included in the study. At the time of data collection, none of the patients admitted during the study period were still hospitalised. However, because of logistical challenges, including ongoing renovations resulting in misplaced records, ward registers containing admission and discharge dates were unavailable, and information on the number of patients excluded because of extended stays beyond the study period was missing. Length of stay in the cohort was 2–357 days, with a median of 73.5 (IQR 49–120) days. Eighty-three patients (25.5%) had an LOS above the 75th percentile (120 days).

### Socio-demographic and clinical characteristics

The socio-demographic and clinical characteristics are presented in Table 1. The highest proportion of patients were in the 25–34-year age group (35.9%). There were 158 (48.5%) females and 168 (51.5%) males; a large proportion was of black race (74.2%). Most were unemployed (84%), and only 36.1% of the unemployed were receiving a social grant. The majority of patients were single (*n* = 270, 82.8%), had an education level above grade 7 (*n* = 205, 87.1%) and had family contact details in their files (*n* = 319, 97.9%). Of the few patients considered for community placement (*n* = 20, 6.2%), median LOS was 1.8 months after the decision to seek community placement had been made and 7 (35%) were discharged to placement facilities while the rest were discharged home.

**TABLE 1:** Socio-demographic and clinical variables (*N* = 326).

Variable	*n*	%	Median	IQR
**Age (years)**				
18–24	83	25.5	-	-
25–34	117	35.9	-	-
35–44	52	16.0	-	-
45+	74	22.7	-	-
**Gender**				
Female	158	48.5	-	-
Male	168	51.5	-	-
**Race**				
Black people	241	74.2	-	-
White people	41	12.6	-	-
Mixed race people	13	4.0	-	-
Asian people	30	9.2	-	-
**Marital status**				
Single	270	82.8	-	-
Married	30	9.2	-	-
Divorced	11	3.4	-	-
Widowed	15	4.6	-	-
**Education group**				
Grade 7 or less	41	12.9	-	-
Grade 8–12	205	64.7	-	-
Post matric	71	22.4	-	-
**Occupation status**				
Unemployed	274	84.0	-	-
Employed	52	16.0	-	-
**Unemployment/grant group**				
Unemployed and no grant	175	63.9	-	-
Unemployed and has grant	99	36.1	-	-
**Family contact details**				
Absent	7	2.1	-	-
Present	319	97.9	-	-
**Patient receiving disability grant?**				
No	244	76.2	-	-
Yes	76	23.8	-	-
**Has placement been considered?**				
No	305	93.8	-	-
Yes	20	6.2	-	-
**Duration of inpatient treatment after placement consideration (in months), median (IQR)**	-	-	1.8	1.0, 5.0
**Number of previous psychiatric admissions**				
Index	84	28.7	-	-
Once or twice	101	34.5	-	-
Three or more times	108	36.9	-	-
**Comorbid psychiatric diagnosis**				
No	217	66.6	-	-
Yes	109	33.4	-	-
**Primary diagnosis**				
Psychotic disorders	157	48.2	-	-
Affective disorders	107	32.8	-	-
Cognitive disorders	19	5.8	-	-
Other	43	13.2	-	-
**Psychotic disorders**				
Schizophrenia	92	58.6	-	-
Schizoaffective	41	26.1	-	-
Other	24	15.3	-	-
**Mental healthcare act admission status**				
Voluntary	35	10.8	-	-
Assisted	14	4.3	-	-
Involuntary	276	84.9	-	-
**Family history of mental illness**				
No	208	69.3	-	-
Yes	92	30.7	-	-

IQR, interquartile range.

Clinical information indicated the majority, 108 (36.9%) patients, had at least three previous admissions. Schizophrenia spectrum and other psychotic disorders were the primary diagnosis in 157 (48.2%) participants. Affective disorders included mood disorders such as bipolar mood and depressive disorders. Other diagnosis included personality disorders, trauma and stressor-related disorders, substance use-related disorder and intellectual disability. The majority of participants (276, 84.9%) were involuntary inpatient admissions.

### Association between length of stay and socio-demographic factors

The associations between LOS and socio-demographic factors are displayed in Table 2. There was no difference in the median age between the SIS and long-stay groups. There was a statistically significant relationship between LOS and gender (*p* < 0.001), race (*p* = 0.001), marital status (*p* = 0.016) and receiving a disability grant if unemployed (*p* < 0.014). Patients in the long-stay group were more likely to be male, black and single. Patients in the long-stay group were more likely to have a scholastic attainment of grade 7 or less (23.1%) compared to those in the SIS group (9.6%). Additionally, the attainment of a post-matric qualification (14.1% vs. 25.1%) and employment (12.0% vs. 17.3%) was less likely in the long-stay group. Placement, a process where the treating clinician transfers patients who require assistance but not active inpatient care to a step-down or sub-acute facility, was less likely to be considered among the SIS group compared to the long-stay group (96.7% and 85.5%, respectively), and this difference was significant (*p* = 0.001).

**TABLE 2:** Association between long-stay and socio-demographic factors.

Variable	SIS (*N* = 243) No				Long-stay hospitalisation (*N* = 83) Yes				*p*	Test
	*n*	%	Median	IQR	*n*	%	Median	IQR		
**Age (years)**	-	-	-	-	-	-	-	-	0.95	Pearson’s Chi-squared
18–24	61	25.1	-	-	22	26.5	-	-	-	-
25–34	87	35.8	-	-	30	36.1	-	-	-	-
35–44	38	15.6	-	-	14	16.9	-	-	-	-
45+	57	23.5	-	-	17	20.5	-	-	-	-
**Gender**	-	-	-	-	-	-	-	-	< 0.001	Pearson’s Chi-squared
Female	135	55.6	-	-	23	27.7	-	-	-	-
Male	108	44.4	-	-	60	72.3	-	-	-	-
**Race**	-	-	-	-	-		-	-	0.001	Fisher’s exact
Black people	167	69.0	-	-	74	89.2	-	-	-	-
White people	37	15.3	-	-	4	4.8	-	-	-	-
Mixed race people	13	5.4	-	-	0	0.0	-	-	-	-
Asian people	25	10.3	-	-	5	6.0	-	-	-	-
**Marital status**	-	-	-	-	-	-	-	-	0.016	Fisher’s exact
Single	193	79.4	-	-	77	92.8	-	-	-	-
Married	26	10.7	-	-	4	4.8	-	-	-	-
Divorced	9	3.7	-	-	2	2.4	-	-	-	-
Widowed	15	6.2	-	-	0	0.0	-	-	-	-
**Education group**	-	-	-	-	-	-	-	-	0.003	Pearson’s Chi-squared
Grade 7 or less	23	9.6	-	-	18	23.1	-	-	-	-
Grade 8–12	156	65.3	-	-	49	62.8	-	-	-	-
Post matric	60	25.1	-	-	11	14.1	-	-	-	-
**Occupation status**	-	-	-	-	-	-	-	-	0.26	Pearson’s Chi-squared
Unemployed	201	82.7	-	-	73	88.0	-	-	-	-
Employed	42	17.3	-	-	10	12.0	-	-	-	-
**Disability grant**	-	-	-	-	-	-	-	-	< 0.001	-
No	196	82.4	-	-	48	58.5	-	-	-	-
Yes	42	17.6	-	-	34	41.5	-	-	-	-
**Unemployed/grant**	-	-	-	-	-	-	-	-	0.014	Pearson’s Chi-squared
Unemployed and no grant	137	68.2	-	-	38	52.1	-	-	-	-
Unemployed and has grant	64	31.8	-	-	35	47.9	-	-	-	-
**Family contact details**	-	-	-	-	-	-	-	-	0.68	Fisher’s exact
Absent	6	2.5	-	-	1	1.2	-	-	-	-
Present	237	97.5	-	-	82	98.8	-	-	-	-
**Has placement been considered?**	-	-	-	-	-	-	-	-	< 0.001	Pearson’s Chi-squared
No	234	96.7	-	-	71	85.5	-	-	-	-
Yes	8	3.3	-	-	12	14.5	-	-	-	-
**Duration of inpatient treatment after placement consideration, median (IQR)**	-	-	1.0	0.5, 2.0	-	-	4.5	1.0, 6.0	0.016	Wilcoxon rank-sum

IQR, interquartile range; SIS, short-intermediate stay.

### Association between length of stay and clinical variables

Associations between LOS and clinical variables is presented in Table 3. Long-stay admissions were significantly associated with diagnosis (*p* < 0.001), admission status (*p* < 0.001) and number of admissions (*p* = 0.01). A primary diagnosis of a psychotic disorder was significantly associated with long-stay (*p* < 0.001). All patients in the long-stay group were involuntary admissions. To further determine association between the number of previous admissions and long-stay, a bivariate logistic regression was conducted and the odds of being in the long-stay group was 2.3 times more for patients who had three or more previous admissions compared to those who had an index admission (*p* = 015; CI [confidence interval] 1.1–4.5). A family history of mental illness was not associated with LOS.

**TABLE 3:** Association between length of stay and clinical variables.

Variable	SIS (*N* = 243)		Long-stay (*N* = 83)		*p*	Test
	*n*	%	*n*	%		
**Number of previous psychiatric admissions**	-	-	-	-	0.010	Fisher’s exact
Index	68	30.9	16	21.9	-	-
Once or twice	82	37.3	19	26.0	-	-
Three or more times	70	31.8	38	52.1	-	-
**Primary diagnosis**	-	-	-	-	< 0.001	Fisher’s exact
Psychotic disorders	92	37.9	65	78.3	-	-
Affective disorders	96	39.5	11	13.3	-	-
Cognitive disorders	16	6.6	3	3.6	-	-
Other	39	16.0	4	4.8	-	-
**Psychosis**	-	-	-	-	0.019	Pearson’s Chi-squared
Schizophrenia	46	50	46	71	-	-
Schizoaffective	27	29	14	22	-	-
Other	19	21	5	8	-	-
**Mental healthcare act admission status**	-	-	-	-	< 0.001	Fisher’s exact
Voluntary	35	14.5	0	0.0	-	-
Assisted	14	5.8	0	0.0	-	-
Involuntary	193	79.8	83	100.0	-	-
**Family history of mental illness**	-	-	-	-	0.30	Pearson’s Chi-squared
No	151	67.7	57	74.0	-	-
Yes	72	32.3	20	26.0	-	-

SIS, short-intermediate stay.

### Regression model of long-stay and socio-demographic and clinical variables

Table 4 shows a regression model of long-stay controlling for socio-demographic and clinical variables. Being male remained significantly associated with long-stay, odds ratio (OR) 2.76 (CI 1.40–5.47) while receiving a disability grant when unemployed approached significance 2.13 (1.00–4.54). Having affective disorders and other disorders was associated with lower odds of long-stay compared to psychotic disorders OR 0.15 (CI 0.06–0.39) and OR 0.11 (CI 0.03–0.40), respectively.

**TABLE 4:** Length of stay controlling for socio-demographic and clinical variables.

Variable	OR	SE	*p*	95% CI	
**Gender**					
Male	2.76	0.96	0.004	1.40	5.47
Female	-	-	-	-	-
**Age (years)**					
25–34	0.73	0.29	0.45	0.33	1.62
35–44	0.66	0.36	0.46	0.22	1.97
45+	0.59	0.33	0.35	0.19	1.77
**Primary Dx [Psychotic d/o]**					
Affective disorders	0.15	0.07	0.00	0.06	0.38
Cognitive disorders	0.30	0.20	0.08	0.08	1.16
Personality disorders and others	0.11	0.07	0.001	0.03	0.39
**Unemployed [No disability grant]**					
Has disability grant	2.13	0.82	0.05	1.00	4.54

OR, odds ratio; SE, standard error; CI, confidence interval.

## Discussion

The study was conducted in a tertiary psychiatric hospital in SA, aiming to determine the duration of long-stay and to describe the socio-demographic and clinical profile of long-stay patients. The LOS varied from 2 to 357 days, with a median stay of two and a half months. Long-stay was determined to be a LOS of 120 or more days and was associated with being male, having a psychotic disorder and a history of multiple previous admissions.

The socio-demographic factors associated with long-stay were being male, being black, being single, receiving a disability grant and having a lower level of education. The findings of a significant association between male gender and long-stay was similar to findings in the UK and Nigeria.^19,23^ Various reasons have been cited in the literature to explain why males are more likely to have longer LOS compared to females. Males are reported to be unlikely to seek mental health treatment early and therefore have longer duration of untreated symptoms,^30^ which may lead to higher symptom severity at presentation. Taiwo, Ladapo^23^ also suggested that males with psychotic disorders may be more difficult to manage in the community compared to females with the same diagnosis. Other studies have reported LOS to be significantly higher for females, but these studies were in private healthcare facilities where the mean LOS was less than 3 weeks, possibly as a consequence of being capped by health insurance providers, and the study population predominantly had been diagnosed with affective disorders.^20,31^ Contrary to findings in this study, Vranda, Ranjith^25^ reported that being female was associated with higher LOS, and their study was set in a tertiary psychiatric hospital. Studies in other settings have also found being black or of minority ethnic groups and a lower socioeconomic status to be associated with longer LOS.^32,33,34^ Reasons may be related to social determinants of health with access to quality care biased against economically disadvantaged individuals. Data from different countries across Europe also show that economic disadvantage is associated with longer hospital stay.^35^ In SA, the legacy of colonisation and apartheid continues to prevail, and this may be a factor contributing to higher LOS in the black racial group.^36^

This study found a predominantly single long-stay population, which is similar to studies across the world, all reporting majority single or unmarried population.^19,20,21,23,24,37^ Being single may be associated with less social support and longer LOS.^38^ Others have reported conflicting findings with regard to being married. Silva et al. found being married to be associated with shorter admissions while Vranda, Ranjith^25^ reported that being married is associated with long-stay. The contradictory findings of the effect of marital status on LOS is in keeping with the observation of both positive and negative effects of marriage on the individual’s mental illness.^39^ Positive effects of being married include better support system and quality of life and reduction of social isolation of homelessness and of suicidality.^39,40^ Difficulties include not disclosing the diagnosis because of fear of rejection, poor adherence, sexual dysfunction because of mental illness or medication side effects, poor marital adjustment and increased risk of relapse.^39^ These effects point to the importance of the presence and the nature of family or social network and stress the need to provide interventions to improve capacity for developing and maintaining social networks. Interventions include, among other things, social skills training, counselling, therapy and providing support.^39,40,41^

Receiving a disability grant was associated with long-stay. In SA, a disability grant is provided for individuals with psychiatric or physical conditions that impair function to the extent that the individual is unfit to work for longer than 6 months.^42^ Long-stay has been associated with functional impairment in other settings as well.^12,22,25^ While functional impairment as a result of mental illness may be a chronic condition, long-stays may be an indication of the health inequities and additional barrier to accessing quality healthcare, as extensively described in literature on people with disabilities.^43^

When the family is unable to cope with the demands of the burden of care for a patient, placement in a community facility for supported living is considered a viable and cost-effective option. Consideration for placement was more likely in patients in the long-stay group compared to the SIS group, this decision was subsequently followed by an average LOS of over 4 months. This may suggest limited availability of community placement options, leading to extended hospital stays for some patients and less efficient use of tertiary care services.

Employment has been described as an important mental health intervention as it fosters self-reliance and allows for a meaningful life.^44^ In our study, unemployment rates were high in the whole cohort and there was no significant difference between the SIS and long-stay groups. Other studies have reported unemployment rates as high as 80% in long-stay patients.^19,21,22,31^

Higher levels of education have been associated with a better prospect of employment.^45^ Although this study found that patients in the long-stay group were more likely to have a lower education level compared to the SIS group, this did not translate to better employment in the relatively better-educated SIS group. In SA, the unemployment rate is reported to be 33%, and this could explain the lack of difference in employment between the groups.^46^ In other settings, however, illiteracy and a low level of education has been associated with LOS.^21,37,47^

Consistent with data from five European countries, clinical factors associated with long-stay in this study were a diagnosis of a psychotic disorder, three or more previous admissions and an involuntary admission status.^35^ A diagnosis of psychosis is reported to be a strong predictor of long-stay in multiple studies.^2,19,21,22,23,25,31,37,41^ An association between LOS and readmission rates has also been reported, as found in this study.^31,48^ Psychotic disorders and readmission rates have both been linked to long-stay. In psychosis, multiple episodes of relapse, as evidenced by higher readmission rates, is a poor prognostic factor. It is therefore important that treatment of psychotic disorders is not only limited to initiation of antipsychotic medications but also includes interventions for relapse prevention^49^ such as long-acting injectable (LAI) antipsychotics, psychoeducation, family therapy and technology-based interventions.^50^

The association of involuntary admission and long-stay is in line with the report of previous studies.^12,47,48,51^ Involuntary admissions enable provision of treatment to patients with mental disorders who are incapable of making informed decisions regarding their need for treatment and are also unwilling to receive the necessary care. The association of long-stay with involuntary admission and psychotic disorders is in keeping with the partial or complete lack of insight, which is a feature in up to 80% of individuals with a chronic psychotic disorder and poor insight being associated with increased LOS in these patients.^52,53^ Evidence suggests that insight in psychosis can be improved.^54^ Therefore, treatment of psychotic disorders should not only focus on symptom management but also include interventions aimed at enhancing insight from early in the course of the illness.

Vranda, Ranjith^25^ found an association between a family history of mental illness and long hospital stay in India, but this association was not observed in this study. This difference may be because of variations in traditional family structures. In India, family units often live in close proximity, with up to three or more generations residing together, facilitating awareness of family medical histories.^55^ In contrast, South African families are often shaped by migrant labour practices, resulting in many children not being raised by their biological parents, and this may affect knowledge of the family history of mental illness.^56^

### Study’s limitations

While the study highlights important factors contributing to lengthy hospital stays, the authors acknowledge a number of limitations. The study was conducted in a hospital that provides tertiary psychiatric services and serves as a referral centre for other hospitals. Therefore, the LOS described in the study did not account for the number of days spent in the referring hospitals, likely underestimating the total in-hospital stay. Nevertheless, these findings can be representative of tertiary psychiatric hospitals that provide similar services in the country. Another factor that may have resulted in undercounting of LOS was that there was no available information regarding the number of patients who would have been hospitalised beyond the study period.

The unavailability of key ward records resulted in the loss of valuable information essential for service delivery. This highlights the challenges associated with paper-based record keeping. The authors echo the recommendation by Marutha and Ngoepe^57^ that electronic systems could offer a more efficient solution for managing patient care information.

The study did not include data on treatment and comorbid medical illnesses and these are factors that could have possibly impacted on LOS. Lastly, retrospective studies have inherent limitations by design, with the quality of findings dependent on the accuracy of clinical records. However, the information collected in this study is likely to be accurate because the hospital is a teaching and specialist training institution, where management of all patients is overseen by senior clinicians.

## Conclusion

This study found that long-stay was associated with psychotic disorders and factors such as male gender, being black, multiple readmissions, involuntary admissions and functional decline. These factors are linked to serious mental illness, particularly psychosis, and may reflect disease severity. The findings emphasise the need for early intervention in psychotic disorders, which drive up mental healthcare costs because of extended hospital stays. Identifying factors linked to prolonged stays helps policymakers and healthcare teams develop programmes that provide efficient care, initiate interventions, improve transitions to outpatient care and support community reintegration.
